# Total Laboratory Automation and Three Shifts Reduce Turnaround Time of Cerebrospinal Fluid Culture Results in the Chinese Clinical Microbiology Laboratory

**DOI:** 10.3389/fcimb.2021.765504

**Published:** 2021-12-02

**Authors:** Weili Zhang, Siying Wu, Jin Deng, Quanfeng Liao, Ya Liu, Li Xiong, Ling Shu, Yu Yuan, Yuling Xiao, Ying Ma, Mei Kang, Dongdong Li, Yi Xie

**Affiliations:** Department of Laboratory Medicine, West China Hospital of Sichuan University, Chengdu, China

**Keywords:** total laboratory automation, turnaround time, clinical microbiology, Kiestra, CSF

## Abstract

**Background:**

Total laboratory automation (TLA) has the potential to reduce specimen processing time, optimize workflow, and decrease turnaround time (TAT). The purpose of this research is to investigate whether the TAT of our laboratory has changed since the adoption of TLA, as well as to optimize laboratory workflow, improve laboratory testing efficiency, and provide better services of clinical diagnosis and treatment.

**Materials and Methods:**

Laboratory data was extracted from our laboratory information system in two 6-month periods: pre-TLA (July to December 2019) and post-TLA (July to December 2020), respectively.

**Results:**

The median TAT for positive cultures decreased significantly from pre-TLA to post-TLA (65.93 *vs* 63.53, *P*<0.001). For different types of cultures, The TAT of CSF changed the most (86.76 *vs* 64.30, *P*=0.007), followed by sputum (64.38 *vs* 61.41, *P*<0.001), urine (52.10 *vs* 49,57, *P*<0.001), blood (68.49 *vs* 66.60, *P*<0.001). For Ascites and Pleural fluid, there was no significant difference (*P*>0.05). Further analysis found that the incidence of broth growth only for pre-TLA was 12.4% (14/133), while for post-TLA, it was 3.4% (4/119). The difference was statistically significant (*P*=0.01). The common isolates from CSF samples were *Cryptococcus neoformans*, coagulase-negative *Staphylococcus, Acinetobacter baumannii*, and *Klebsiella pneumonia.*

**Conclusion:**

Using TLA and setting up three shifts shortened the TAT of our clinical microbiology laboratory, especially for CSF samples.

## Background

Automation has become increasingly common in clinical laboratories, but not yet in clinical microbiology laboratories. There are many possible reasons, such as diversity of clinical samples, culture media, and culture conditions (Dauwalder et al., 2016). The traditional manual operation of the clinical microbiology laboratory can be time-consuming and inefficient. For example, around 45 manual steps are required from specimen receiving to final report (Croxatto et al., 2015). Now the blood culture instrument adopts a continuous monitoring function to shorten the time of bloodstream infection diagnosis and treatment, which inspires people to explore the development of an automated system of clinical microbiology laboratories. Total laboratory automation (TLA) in clinical microbiology laboratories is defined as instrumentation that automates the bacteriology processes from specimen receiving to discarding plates (Thomson and McElvania, 2019). There are two widely used automation systems available: BD Kiestra Total Laboratory Automation (TLA; Becton-Dickinson, Sparks, MD) and Copan WASPLab (Copan Diagnostics Inc, Murrieta, CA) (Dauwalder et al., 2016; Croxatto et al., 2016; Thomson and McElvania, 2019).

Our clinical microbiology laboratory implemented the BD Kiestra TLA platform from March 2020. To make the best out of the advantages of TLA continuous reception, regular photography, and reading images *via* the computer screen, we have adjusted the schedule of working shifts from one type (day shift) to three types (morning shift, day shift, and night shift). The purpose of this research is to investigate whether the turnaround time (TAT) of our laboratory has changed since the adoption of TLA, as well as to optimize laboratory workflow, improve laboratory testing efficiency, and provide better services for clinical diagnosis and treatment.

## Materials and Methods

This study was performed in West China Hospital of Sichuan University with a total of 4300 beds. The clinical microbiology laboratory performs approximately 23000 cultures per month.

Before and after using TLA, our inoculation process for each type of specimen was the same, that was, using the same material plates (Antubio, Zhengzhou, China) and the same amount of inoculation (for example, for urine, inoculating 10ul sample on the blood plate and MacConkey plate). The diameter of plates varied from 7cm before TLA to 9cm after TLA to meet the requirements of Kiestra TLA.

### Pre-TLA Process

**Figure 1:**
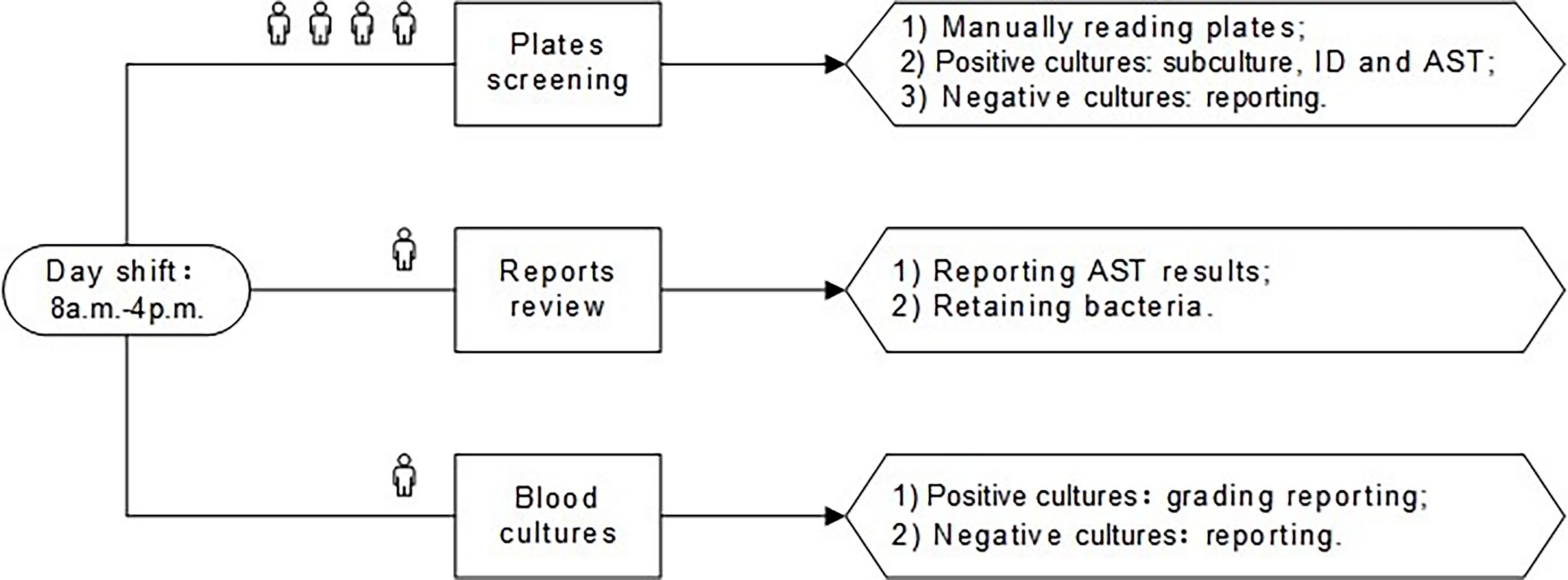
The day shift of pre-TLA: four people for plates screening, one person for reports review, and one person for blood cultures. ID, identification; AST, antimicrobial susceptibility testing; TLA, total laboratory automation.

Inpatient and outpatient specimens were received for processing throughout the day. For the conventional plating technique, specimens were manually streaked using corresponding agar plates and manually placed into incubators. Plates were incubated overnight, and then manually removed from the incubators and read during the day shift (8 a.m. to 4 p.m.). The main work of the plates screening post was to manually remove the plates from the incubator for reading, perform subcultures, identification (ID), and antimicrobial susceptibility testing (AST) for the culture-positive specimens, and report the culture-negative specimens. The reports review post was to report AST results and retain bacteria. The blood cultures post was required to grade reports of positive blood cultures and report negative results. The day shift pre-TLA of our lab was elaborated in **Figure 1**.

### Post-TLA Process and Three Shifts

**Figure 2:**
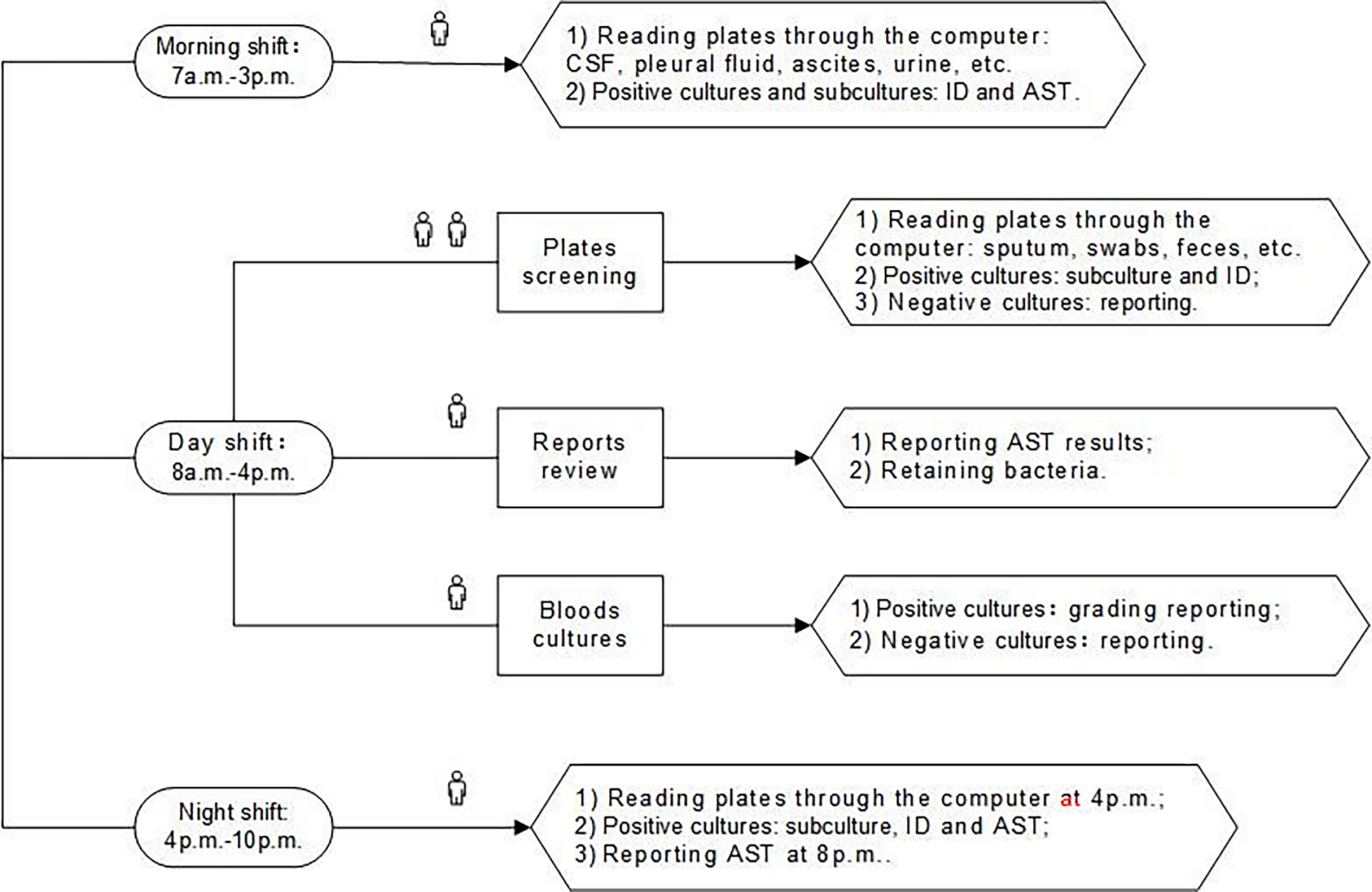
The three shifts of post-TLA: one person for morning shift, four people for day shift, and one person for night shift. CSF, cerebrospinal fluid; ID, identification; AST, antimicrobial susceptibility testing; TLA, total laboratory automation.

BD Kiestra TLA was implemented in March 2020. With TLA, specimens were inoculated onto corresponding agar plates by the fully automated InoqulA+ system, and plates were transported *via* conveyor tracks (ProceedA) to the incubators (ReadA Compact). Digital images of the plates were taken at the user-specified time (every 6 h after reception) and were interpreted by technologists throughout the three shifts (morning shift: 7 a.m.-3 p.m., day shift: 8 a.m.-4 p.m., night shift: 4 p.m.-10 p.m.) whenever images became available. The staff in morning shift were mainly responsible for the ID and AST of the positive cultures with a single pure colony cultured from cerebrospinal fluid (CSF), pleural fluid, ascites, urine, and other specimens, as well as the subcultures isolated from the positive cultures of the previous day. The staff in day shift was responsible for the review of AST reports, the subculture, and identification of sputum, swabs, feces, and other specimens. The night shift started the second round of plates screening and subsequent processing at 4 p.m. and then the second round of AST reports review at 8 p.m. The three shifts post-TLA of our lab were elaborated in **Figure 2**.

### Laboratory Process Flow of CSF Specimens

**Figure 3:**
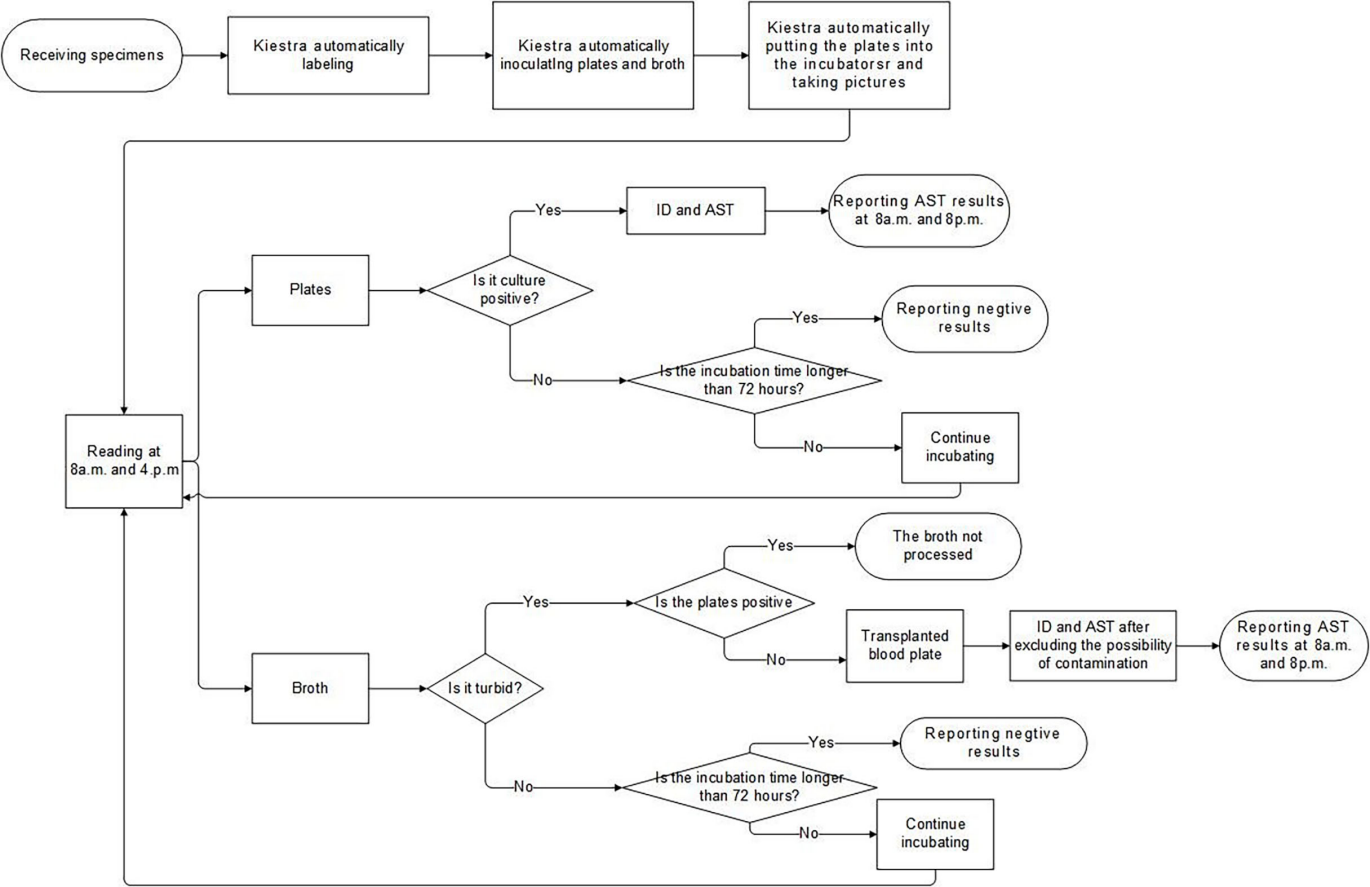
The Laboratory process flow of CSF specimens. ID, identification; AST, antimicrobial susceptibility testing; CSF, cerebrospinal fluid.

As with other types of specimens, when the system received sample information of CSF, the Kiestra automatically generated the corresponding plates: blood agar (BA) plates, chocolate agar (CA) plates, and thioglycollate broth. The CSF samples were handled semi-automated mode, and 50 μl of CSF was inoculated separately on each plate or the broth, which was the same as pre-TLA. Then the plates were automatically put into the incubators and taken pictures at regular intervals. At 8 a.m. and 4. p.m., we read the images of the plates through the computer, and proceeded to the next step for the culture-positive specimens, while the culture-negative specimens were reported with negative results after 72 hours. At the same time, we would also observe the growth of broth. If the broth and the agar plates were positive, we directly performed ID and AST on the plates without further processing the broth. If the broth was positive and the agar plates were negative, we transferred the broth to the blood agar plate for subculture and then performed ID and AST after ruling out the possibility of contamination. The process flow of CSF specimens of our lab was elaborated in **Figure 3**.

### MALDI-TOF MS ID and AST

Colonies were identified using the Bruker MALDI Biotyper instrument (Bruker Daltonik GmbH, Bremen, Germany). Species identification was considered valid if scores ≥2.0. Scores <1.7 were regarded as unreliable; scores ≥1.7 and <2.0 were considered as a reliable identification of genus.

Colonies from primary cultures or subcultures were selected for AST by Kirby-Bauer disk diffusion (KB) (ThermoFisher, Massachusetts, USA) and Vitek II (BioMérieux, Montréal, France) as per CLSI (CLSI, 2019, 2020). For Vitek II susceptibility testing, colonies were picked and suspended in 0.45% saline. Turbidity of the inoculum was adjusted using the densitometer and the Vitek GN04 and GN67 AST cards were used for Gram-negative, Vitek GP67 for Gram-positive pathogens, or Vitek GP68 for *Streptococcus pneumoniae*, respectively. For KB testing, a 0.5 McFarland standard inoculum was prepared by suspending isolated colonies from the primary BA plate incubated. Antibiotic disks were dispensed on the inoculated Mueller-Hinton agar (MHA) (Antubio, Zhengzhou, China) and incubated at 35°C according to CLSI testing and interpretation recommendations (CLSI, 2019, 2020).

### Data Extraction and Analysis

The data including the time node of the sample reception, processed, and et al, was generated or recorded by our Laboratory Information System (Xinhe, Shanghai, China) spontaneously. We can export the detailed information, helping us to optimize laboratory management. To evaluate the impact of TLA on overall TAT, laboratory data for all cultures was extracted from our laboratory information system in two 6-month periods: pre-TLA (July to December 2019) and post-TLA (July to December 2020), respectively. During both periods, MALDI-TOF MS was used for the ID of isolates. TAT of positive cultures was defined as the time from specimen receipt by the laboratory to the final report. Comparisons of TAT in pre-TLA and post-TLA periods were analyzed using the Mann-Whitney U test and Kruskal-Wallis tests, where applicable. All analyses performed are two-tailed, and *P* values of <0.05 were considered significant.

## Results

### Comparison of the Time Needed From Receiving the Specimens to Final Reports Between Pre-TLA and Post-TLA Periods

**Figure 4:**
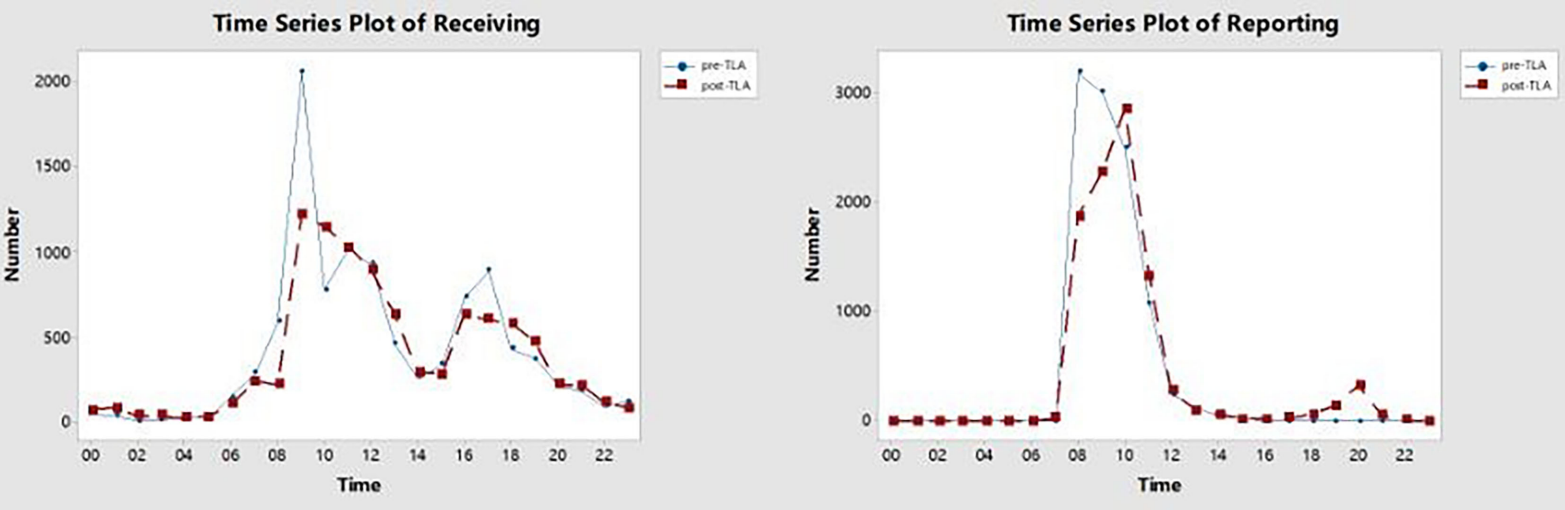
The time series plot of receiving and reporting in pre-TLA and post-TLA. The X-axis represents the time in a 24-hour time display, and the Y-axis represents the number of receiving or reporting at each moment. TLA, total laboratory automation.

**Figure 4** shows that there were two peaks of specimen reception starting at 9 a.m. and 5 p.m. After using Kiestra, the curve of the number of specimens received over time was flatter. Before TLA, the review of AST reports mostly started at 8 a.m. After using Kiestra, there was a small peak for AST report review at 8 p.m., because we scheduled the night shift.

### Comparison of TAT of Different Specimen Types Between Pre-TLA and Post-TLA Periods

**Figure 5:**
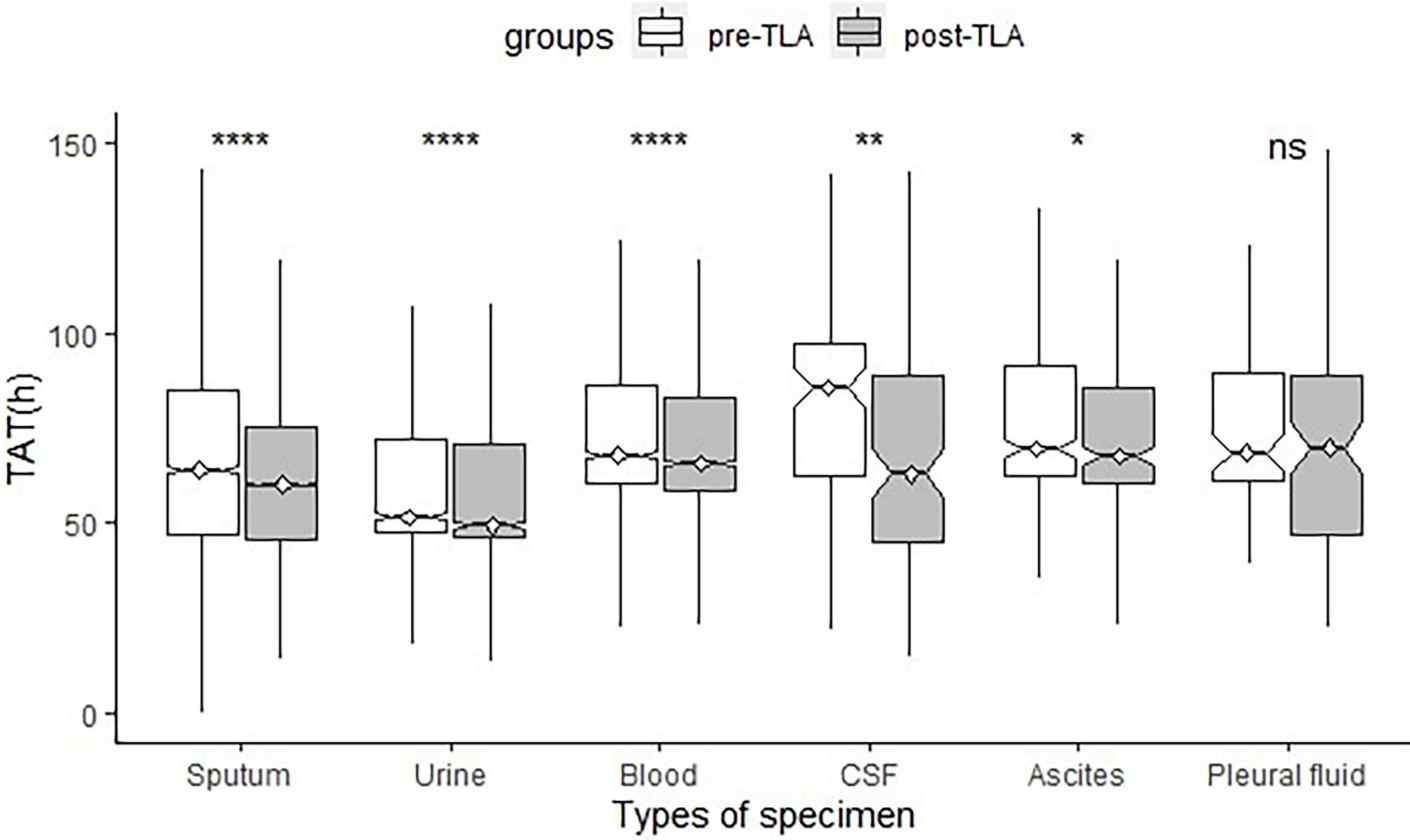
Comparison of TAT of different specimen types between pre-TLA and post-TLA. ****P < 0.0001; **P < 0.01; *P < 0.1; ns, no significance; TLA, total laboratory automation; TAT, turnaround time; CSF, cerebrospinal fluid.

A total of 29199 positive-culture specimens were analyzed (14956 and 14243 from the pre-TLA and post-TLA periods, respectively) (**Table 1** and **Figure 5**). For AST reports of all types of specimens, the median TAT for positive cultures decreased significantly from pre-TLA to post-TLA (65.93 *vs* 63.53, *P*<0.001). For different types of cultures, The TAT of CSF changed the most (86.76 *vs* 64.30, *P*=0.007), followed by sputum (64.38 *vs* 61.41, *P*<0.001), urine (52.10 *vs* 49,57, *P*<0.001), blood (68.49 *vs* 66.60, *P*<0.001). For Ascites and Pleural fluid, there was no significant difference (*P*>0.05).

**Table 1:** Comparison of TAT between pre-TLA and post-TLA.

Culture types	No. of positive-culture (n)		TAT (h)		*P*-value
	Pre-TLA	Post-TLA	Pre-TLA	Post-TLA	
**Total**	68127	69126	65.93 (47.91-86.30)	63.53 (46.85-77.37)	<0.001
**Sputum**	7444	6312	64.38 (47.00-86.50)	61.41 (46.03-79.08)	<0.001
**Urine**	1772	2109	52.10 (47.80-72.02)	49.57 (46.43-71.00)	<0.001
**Blood**	2329	2408	68.49 (60.72-87.43)	66.60 (58.99-85.17)	<0.001
**CSF**	113	119	86.76 (62.37-109.81)	64.30 (45.13-96.42)	0.007
**Ascites**	285	278	69.55 (62.38-91.79)	68.16 (60.84-86.95)	0.054
**Pleural fluid**	97	107	68.83 (61.05-92.44)	69.97 (46.79-91.55)	0.325

Results were shown as the median TAT in hours (with interquartile range in parentheses) and used the Mann-Whitney U test. TLA, total laboratory automation; TAT, turnaround time; CSF, cerebrospinal fluid.

### Comparison of the Incidence of Broth Growth Only Between Pre-TLA and Post-TLA

Further analysis found that the incidence of broth growth only for pre-TLA was 12.4% (14/133), while for post-TLA, it was 3.4% (4/119). The difference was statistically significant (*P*=0.01). When we excluded these specimens, the median TAT of pre-TLA reduced from 86.76 to 70.25 hours, while the median TAT of post-TLA reduced from 64.30 to 63.71 hours. The *P*-value estimating the difference between the two periods also changed from 0.007 to 0.013.

### Distribution of Strains Isolated From CSF

**Figure 6:**
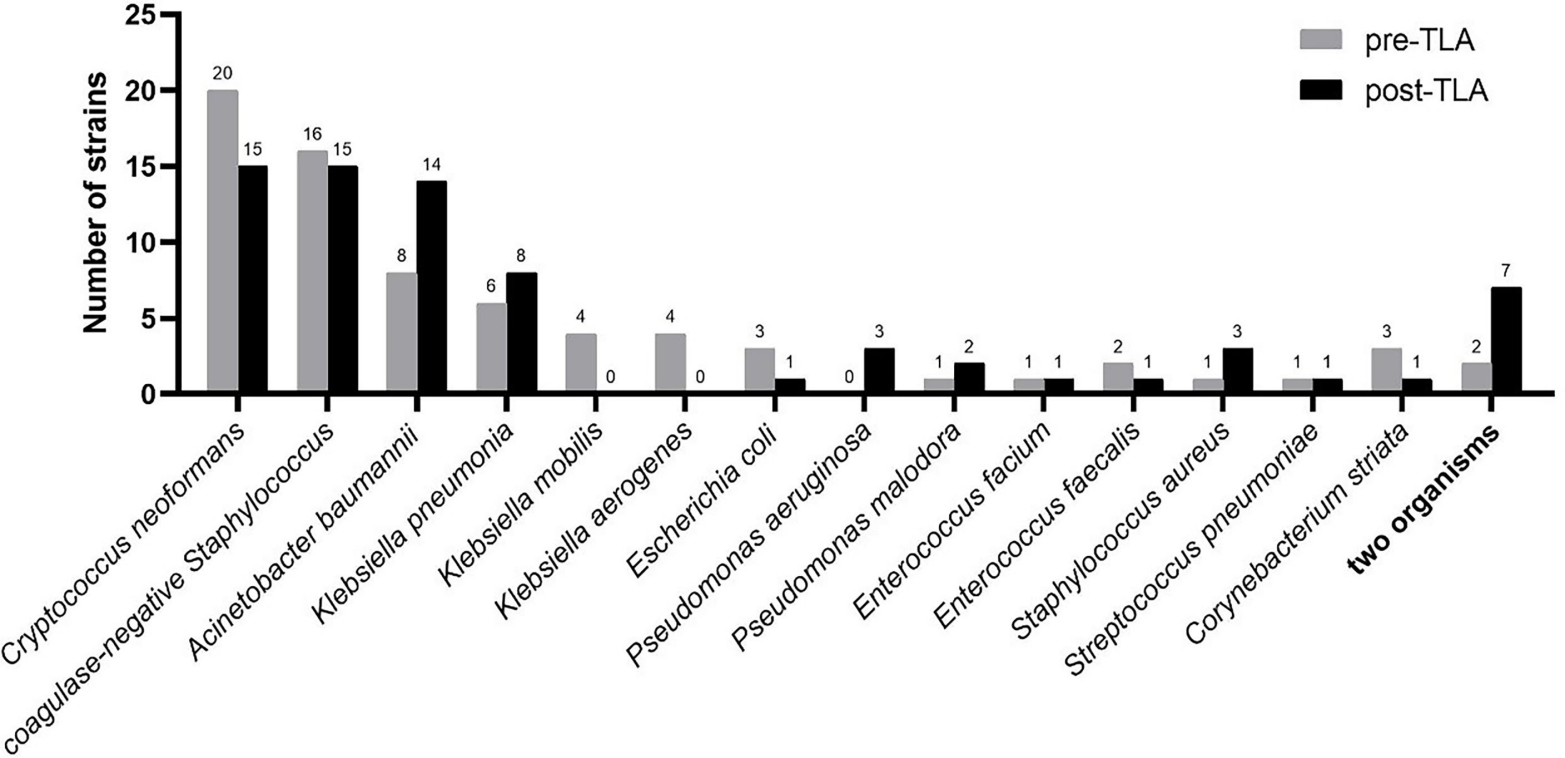
Distribution of strains isolated from CSF in pre-TLA and post-TLA. CSF, cerebrospinal fluid; TLA, total laboratory automation.

**Figure 6** shows the distribution of strains isolated from CSF (only one strain was not shown). The common isolates of CSF specimens were *Cryptococcus neoformans*, coagulase-negative *Staphylococcus, Acinetobacter baumannii*, and *Klebsiella pneumonia*, which respectively accounted for 20.8%, 18.5%, 13.1%, 8.3%.

## Discussion

We deeply modified the work organization of laboratory technicians and our schedule, which made our two compared study periods not strictly comparable because there were two variables: TLA and three shifts, but automation implementation in the lab need to perform these modifications in lab organization, notably reading time for the plates. Thanks to automation, it is quite easy to add additional reading time points as in our lab that plates were imaged every 6 hours. We did not reduce the number of laboratory technicians during the two periods. Thus, we modified the laboratory technicians’ schedule to increase reading, ID, AST, etc. times and it was the combination of TLA and an extended reading time of laboratory technicians schedule that allowed a decrease of TAT. Automation helped to increase the duration of lab service without additional laboratory technicians. Therefore, like other studies, our lab also benefited from savings in labor cost (Culbreath et al., 2021; Zimmermann, 2021) and better control of the costs with reduced TAT thus resulting in a faster diagnosis (Leo et al., 2020). At the same time, reading the plates through the image can increase laboratory biosafety compared to reading the plates directly. Furthermore, if our lab was opened 24/7 with a reading service combined with machine learning (Bailey et al., 2019; Ford and McElvania, 2020; Culbreath et al., 2021), the TAT will perhaps more decrease.

Compared with the pre-TLA, the reception curve of the post-TLA was flatter, which was attributed to the fact that TLA can complete the tasks of receipt, streaking, and incubation of specimens in continuous or random mode rather than only in batch mode. Although this change may not be directly reflected in TAT, it will add the actual incubation time of the plate, increasing culture efficiency (Dauwalder et al., 2016; Graham et al., 2016). After adding a night shift, there was a small peak at 8 p.m. in the reporting curve of the post-TLA, which shortened the TAT by 12 hours and made the report available to clinicians in the next morning, so that the correct antibiotic treatment can be carried out in time (Graham et al., 2016).

Many studies have shown that TLA has the potential to reduce specimen processing time, optimize workflow, and decrease TAT (Mutters et al., 2014; Yarbrough et al., 2018; Theparee et al., 2018; Klein et al., 2018; Bailey and Burnham, 2019). In our study, TAT for all culture-positive specimens and different types of specimens was both reduced. Especially the TAT of CSF was shortened by about 22 hours, which was remarkable. Therefore, we carefully studied the process of our laboratory to determine the cause of the huge change in TAT. Further analysis found that the incidence of broth growth only in the pre-TLA period was significantly higher than that in the post-TLA period. When we excluded these specimens, the TAT difference between the two groups was reduced from 22 hours to less than 7 hours. According to the traditional laboratory process, when it was found that the plates did not grow but the broth grew, the broth was transferred to the plate until the bacteria grew on the plate before the next step of ID and AST, so the TAT could be as long as several days. Therefore, TLA has greatly improved the laboratory culture efficiency of sterile body fluids represented by CSF, enabling patients to receive timely antimicrobial treatment and reducing hospitalization costs and mortality.

Some scholars believe that enrichment broth cultures rarely contribute to the diagnosis of bacterial meningitis, and due to its high false-positive rate, positive CSF enrichment broth cultures contribute to diagnostic uncertainty (Chaudhry et al., 2011; Shah et al., 2012). This also reminds us that if TLA could greatly reduce the proportion of broth growth alone, maybe we can cancel broth enrichment culture, which certainly requires the support of larger experimental data combined with clinical practice.

The most common fungus isolated from CSF was *Cryptococcus neoformans*, and the most common bacteria were coagulase-negative *Staphylococcus*, *Acinetobacter baumannii*, and *Klebsiella pneumonia*, which was consistent with the results of other studies (Gonzalez et al., 2018; Chang et al., 2018; Tian et al., 2019). Some studies have reported that *Alloscardivia omnicolens*, *Gardnerella vaginalis*, *Actinomyces* spp., and *Actinotignum schaalii* were significantly more abundant in the urine samples incubated and processed with TLA (Klein et al., 2018), but we did not find similar pathogen spectrum changes in CSF samples.

In summary, using TLA and setting up three shifts shortened the TAT of our clinical microbiology laboratory, especially for CSF samples. One of the reasons is that the use of TLA increased the efficiency of culture, so the proportion of broth growth only was significantly reduced, which suggested the possibility that broth enrichment is not necessary when using TLA. Our research has some limitations. We conducted a retrospective study and derived data from Laboratory Information System, which may l present inaccurate results. Larger sample size will also be required for further research since the number of culture-positive samples isolated from CSF was relatively small in our current study. Finally, the “pre” and “post” timeframes of this study were also “pre” and “post” of COVID-19. it was difficult for us to assess whether and how much the COVID-19 affected the clinical microbiology laboratory process.

## Data Availability Statement

The datasets presented in this article are not readily available to comply with the Institutional data management requirements. Requests to access the datasets should be directed to Weili Zhang, 648608511@qq.com.

## Author Contributions

WZ: Data analysis(lead); Writing-original draft (lead). SW and JD: Methodology (lead). QL, LX, LS, and YY: Methodology (supporting). YL and YLX: Data analysis (supporting). YM and MK: Writing-review & editing (supporting). DL and YX: Writing-review & editing (lead). All authors contributed to the article and approved the submitted version.

## Funding

This work was supported by the Special Foundation for National Science and Technology Basic Research Program of China (Grant No. 2019FY101200).

## Conflict of Interest

The authors declare that the research was conducted in the absence of any commercial or financial relationships that could be construed as a potential conflict of interest.

## Publisher’s Note

All claims expressed in this article are solely those of the authors and do not necessarily represent those of their affiliated organizations, or those of the publisher, the editors and the reviewers. Any product that may be evaluated in this article, or claim that may be made by its manufacturer, is not guaranteed or endorsed by the publisher.
